# Estimation of postmortem interval using the data of insulin level in the cadaver׳s blood

**DOI:** 10.1016/j.dib.2016.02.059

**Published:** 2016-03-02

**Authors:** Sachil Kumar, Anoop K. Verma

**Affiliations:** a Post Graduate Department of Pathology, K.G. Medical University, Lucknow, UP, India; b Department of Forensic Medicine & Toxicology, K.G. Medical University, Lucknow, UP, India

**Keywords:** Blood, Insulin, Post-mortem interval, Cadavers

## Abstract

An assessment of levels of Insulin in cadaveric fluids, to estimate the postmortem interval (PMI) was carried out.

To profile postmortem changes of Insulin, it was extracted at different intervals i.e. (0, 3, 6, 12, 24 h), from the heart of 22 human cadavers. The cases included were the subjects of accidental deaths without any prior history of disease and their exact time of death was known. Immunoanalyzer Cobas e-411 instrument was used to detect the relationship between the amount of Insulin and PMI.

Level of Insulin was measured in cardiac blood. Statically, significant correlations between levels of Insulin and PMI were studied and correlation coefficients were calculated. SPSS (version 12.0) was used for statistical analysis.

Insulin levels in cadaver blood are correlated significantly with PMI with a *p* value of <0.001. When insulin level increases by 1 unit the duration decreases by 0.93 units. The least square regression line is: **[Duration(Y)=22.71−0.93 Insulin level (X)]**

**Specifications Table**

**Table:** 

**Subject area**	Forensic Medicine
**More specific subject area**	Postmortem Interval
**Type of data**	Tables
**How data was acquired**	Blood from human cadavers
**Data format**	Analyzed
**Experimental factors**	Blood from cadavers
**Experimental features**	Insulin level at different time interval
**Geographical Location**	North India
**Data accessibility**	Data is within this article

**Value of the data**

1. Insulin level is useful in the determination of the early post-mortem interval (PMI).
2. Insulin levels in cadaver blood are correlated significantly with PMI with a *p* value of <0.001.
3. Overall, determination of insulin level from postmortem blood offers advantages such an early PMI, cost efficiency and a rapid method.

**Data**

In the last 60 years numerous methods have been proposed for the estimation of the time since death by chemical measures [1], [2], [3], [4], [5], [6]. The data in the present study demonstrate (Supplementary Tables 1–4) that this technique may be a major advance in the determination of the PMI. Statistically, significant correlations between levels of Insulin and PMI were calculated. Insulin levels in cadaver blood are correlated significantly with PMI with a *p* value of <0.001. When insulin level increases by 1 unit the duration decreases by 0.93 units.

## 1 Experimental design, materials and methods

### 1.1 Experimental set up and sample pre-treatment

Blood was collected from death cases of known PMI at the mortuary of King George׳s Medical University, Lucknow, India. From selected cadavers׳ blood was aspirated from the heart after admission of the body to the morgue when autopsy has to be performed and when the body has to be removed by a mortician. All samples were taken immediately to the hospital laboratory where the blood was centrifuged and serum was removed. If the specimens were collected during the working day they were analyzed immediately. Otherwise they were refrigerated in stopper tubes at −18 °C to prevent degradation. The cases selected for study from the medico legal examiners were quite limited and considered primarily of individuals showing no antemortem evidence of disease and who died rapidly from traumatic injuries where the exact time of death was known. The total number of collected samples was 22.

### 1.2 Chemicals and instrumentation

Reagents for the determination of insulin level by an Immunoanalyzer Cobas e-411 instrument were obtained from Roche Diagnostics. The measurement range for the insulin contained in the serum is as follows: 0.200–1000 µU/mL or 1.39–6945 pmol/L. Lowest detection limit was: 0.200 µU/mL (1.39 pmol/L).

### 1.3 Statistical analysis

The results are presented in mean±SD and percentages. The one way analysis of variance [ANOVA] was used to compare the means among the groups. The *p*-value <0.05 was considered significant. All the analysis was carried out by using SPSS 16.0 version [Chicago, Inc., USA].

#### 1.3.1 Ethical approval

Ethical approval declared from the university ethics committee wide letter no-865/R-Cell-12. Ref. code: 55 E.C.M.II A/P20.

## Footnotes

Appendix A Supplementary data associated with this article can be found in the online version at doi:10.1016/j.dib.2016.02.059.
