# Supplementary material for: Estimation of postmortem interval using the data of insulin level in the cadaver׳s blood
Source: Data Brief. 2016 Mar 2;7:354–6. doi: 10.1016/j.dib.2016.02.059 (PMC4781973; doi:10.1016/j.dib.2016.02.059)
Supplement: Supplementary file 1 — Supplementary material [file mmc1.docx]

**Conflict of interest:**

**None**
