# Supplementary material for: Estimation of postmortem interval using the data of insulin level in the cadaver׳s blood
Source: Data Brief. 2016 Mar 2;7:354–6. doi: 10.1016/j.dib.2016.02.059 (PMC4781973; doi:10.1016/j.dib.2016.02.059)
Supplement: Supplementary file 2 — Supplementary material [file mmc2.docx]

| Time since death (hrs) | Insulin level (uU/mL) |
| --- | --- |
| 4 | 23.05 |
| 4.30 | 22 |
| 5 | 21.01 |
| 5.20 | 21.49 |
| 5.45 | 20 |
| 6.45 | 16.87 |
| 7.30 | 14.79 |
| 8 | 12.05 |
| 8.35 | 9.45 |
| 10 | 8.89 |
| 11 | 8.15 |
| 12.15 | 7.08 |
| 14 | 6.45 |
| 17 | 3.58 |
| 19 | 2.40 |
| 21.50 | 1.58 |
| 23.35 | 0.896 |
| 24 | 0.757 |
| 25.15 | 0.512 |
| 26 | 0.379 |
| 26.30 | 0.200 |
| 27 and above | 0.200< |

Table 1: Value of insulin at different post-mortem interval
