# Supplementary material for: Estimation of postmortem interval using the data of insulin level in the cadaver׳s blood
Source: Data Brief. 2016 Mar 2;7:354–6. doi: 10.1016/j.dib.2016.02.059 (PMC4781973; doi:10.1016/j.dib.2016.02.059)
Supplement: Supplementary file 3 — Supplementary material [file mmc3.docx]

Supplementary Table 2: Regression statistics

| Regression statistics | |
| --- | --- |
| Multiple R | 0.930791184 |
| R square | 0.866372228 |
| Adjusted R square | 0.859690839 |
| Standard Error | 3.155060466 |
| Observations | 22 |
