# Supplementary material for: Estimation of postmortem interval using the data of insulin level in the cadaver׳s blood
Source: Data Brief. 2016 Mar 2;7:354–6. doi: 10.1016/j.dib.2016.02.059 (PMC4781973; doi:10.1016/j.dib.2016.02.059)
Supplement: Supplementary file 4 — Supplementary material [file mmc4.docx]

Supplementary Table 3: ANOVA

|  | Df | SS | MS | F | Significance F |
| --- | --- | --- | --- | --- | --- |
| Regression | 1 | 1290.783 | 1290.783 | 129.6695 | 3.41E-10 |
| Residual | 20 | 199.0881 | 9.954407 |  |  |
| Total | 21 | 1489.871 |  |  |  |
