# Supplementary material for: Estimation of postmortem interval using the data of insulin level in the cadaver׳s blood
Source: Data Brief. 2016 Mar 2;7:354–6. doi: 10.1016/j.dib.2016.02.059 (PMC4781973; doi:10.1016/j.dib.2016.02.059)
Supplement: Supplementary file 5 — Supplementary material [file mmc5.docx]

Supplementary Table 4: t-stat

|  | Coefficients | Standard error | t-stat | p-value | Lower 95% | Upper 95% |
| --- | --- | --- | --- | --- | --- | --- |
| Intercept | 22.71987679 | 1.011768 | 22.45562 | <0.001 | 20.60937 | 24.83039 |
| Insulin level | -0.938363166 | 0.082405 | -11.3873 | <0.001 | -1.11026 | -0.76647 |
